# Challenging Institutional Norms to Improve Local-Level Policy for Health and Health Equity: Comment on "Health Promotion at Local Level in Norway: The Use of Public Health Coordinators and Health Overviews to Promote Fair Distribution Among Social Groups"

**DOI:** 10.15171/ijhpm.2018.67

**Published:** 2018-08-04

**Authors:** Matthew Fisher

**Affiliations:** Southgate Institute for Health, Society and Equity, Flinders University, Adelaide, SA, Australia.

**Keywords:** Norway, Public Policy, Health Equity, Community Development, Local Government

## Abstract

The article by Susanne Hagen and colleagues on Health Promotion at Local Level in Norway discusses actions by municipal governments to assess and address heath inequities within their respective regions, as required under the Norwegian Public Health Act (PHA). Although the broad intent of the Norwegian government is to encourage action on social determinants of health (SDH), Hagen et al find that many of the initiatives undertaken by municipalities ‘tend to cash out as single, targeted initiatives,’ and focus on individual behaviours. In this commentary, I use the concept of place-based policy and ideas from policy theory on the institutional behaviours of public policy agencies and services, to discuss reasons behind this narrowing of perspective and policy action. I argue in favour of an alternative approach involving public agencies and services supporting processes of community-led action and social change.

Scandinavian countries are seen as leaders in delivery of social democratic policies to improve public health and health equity through action on social determinants of health (SDH). Norway’s commitments to health equity are a case in point, and there is much that neoliberal nations such as Australia – my home – could learn from their example. The article by Susanne Hagen and colleagues on *Health Promotion at Local Level in Norway*^1^ is of interest because it explores one key part of Norway’s public policy approach; implementation of a *Public Health Act* (PHA) that aims to further health equity gains by stimulating action on SDH and health equity at the local level. In particular, as we learn from the article, the Act requires municipal governments to understand SDH and health inequities in their region by developing a health overview, and to take action by appointing Public Health Coordinators and considering equity in policy decisions and health promotion initiatives. Norwegian municipal governments would seem to have some scope for action on SDH to promote health equity, being responsible for services in ‘primary health care, schooling, care for children and the elderly, social support and services, culture, agriculture, and socio-economic development’ (p. 2). Hagen et al show that health overviews are an important mechanism to stimulate increased municipal government focus and policy action on health equity. However in this commentary my aim is not to review the research in detail but to discuss one particular issue raised in the findings, drawing on thoughts arising from my own research, with colleagues, on health and public policy in Australia^2,3^ : issues concerning *how* public policy action on SDH and health equity at the local level is actually understood and subsequently implemented.

The implementation of the PHA as described by Hagen et al is related to theory, evidence and action on inter-sectoral or whole-of-government approaches to public policy to improve public health or reduce inequities. Such approaches include Health in All Policies^4^ and ‘place-based’ policy.^5^ The former tends to emphasise policy collaboration between government departments; the latter, collaboration between publically-funded services within geographic regions or more localised spaces.^6^ It is the idea of place-based or geographically localised policy action for public health and health equity that I would like to examine here, in relation to the approaches of Norwegian municipal governments.

Characteristics of place-based approaches described in the literature clearly relate to both the Norwegian PHA and related municipal strategies, and a number of themes of interest to public health including: devolution of control and resources to governance structures operating at a regional or local scale, including local governments; tailoring of strategies to suit local conditions; collaboration between publically-funded services; equity considerations; supportive national policy; community development; and asset-based approaches.^5,7,8^ However, in contemplating the merits and potential of place-based approaches for public health, it is important to consider how such ideas are translated into action, via structures and processes of policy implementation^9^ (as I am sure Hagen et al would agree). Smith’s research^10^ has shown that public health researchers’ and advocates’ ideas about appropriate policy action for health can be ‘reinterpreted’ by policy actors to suit their perceived institutional norms and constraints. Furthermore, the structures and practices of institutions such as government departments or municipal governments already embody their own tacit suppositions about policy problems and what is to be done about them.^11^

The problem as I see it, is that when the idea of place-based or localised policy action for public health and health equity is implemented, it frequently resolves into a form of action that is operationally conventional (and thus ‘comfortable’) both for government agencies and social service providers, but not necessarily the most effective for real and durable gains in health and wellbeing. This form of action I would describe in general terms as ‘service provision,’ involving design, delivery and measurement of localised *interventions* notionally intended to improve the health, health behaviours or life skills of the individuals receiving those programs or services. If there is a focus on equity, then such interventions are readily tailored for and targeted toward population groups deemed to be deficient or disadvantaged in one form or another. The organisations delivering these services may meet together to ensure that ‘clients’ have access to the services they are deemed to need.^3^ This description would seem consistent with Hagen and colleagues’ finding that ‘municipalities focusing on equity in health are strengthening their competence to act, but that policy responses so far tend to cash out as single, targeted initiatives’ with a focus on individual behaviour (p. 8).

Such forms of action are not unimportant; they may provide people with access to services that are useful in improving their lives. However, the essential limitation of such action in many cases, I believe, is that it marginalises or overlooks the possibilities for another kind of action that is far less about ‘interventions’ per sé and more about social change; actions in which community members are empowered as leaders, decision makers, and active producers of action rather than merely ‘clients,’ ‘consumers,’ ‘patients’ or members of ‘disadvantaged’ or ‘at risk’ target groups.^12^ In Australia, Aboriginal and Torres Strait Islander groups have led recent arguments in favour of approaches that make this crucial shift from interventions to empowerment at a regional level.^13^ With this approach in view, it is necessary to recognise and understand SDH not only as material conditions but also in terms of psychosocial conditions enabling people to experience a sense of control over the conditions of life,^14^ and strengthen supportive social relationships within their communities.^15,16^ Such actions are necessarily defined locally but include possibilities such as development of local business and employment opportunities, improving community amenity and food security, or building networks for social support. In such ways, community members can engage in actions that improve their lives in meaningful ways, and also are likely to improve their health.^17,18^

However, such approaches are likely to be *uncomfortable* for government agencies and publically funded service providers for three reasons. Firstly, they involve those organisations *not* directing or being in control of what is done, but finding ways to catalyse and/or support community-led actions; actions which may also need time and space to unfold. This can appear as anathema to government ‘needs’ for defined programs, prescriptive accountability and measurement of outputs; and to service agency interests in securing funding for and justifying their own activities. Secondly, a community empowerment approach is not really about ‘delivery’ of a time-bound program but about a longer-term process of social change, in which community members self-organise and choose to reclaim and exercise control over areas of their lives and experience that are important to them. Thirdly, and most importantly, community empowerment approaches to localised action involve and develop people as capable agents in their own lives, not merely as passive consumers of goods and services whether from the public or the private sector. This is anathema to public policy approaches that view people through a narrow lens of disadvantage, deficit or illness.

Findings from the research of Hagen et al suggest that a significant proportion of the activity generated by the PHA at municipal government level to address SDH and health inequities is following the conventional public institutional norms of implementing ‘interventions’ targeted toward individuals. Thus, Norwegian municipal governments and services would appear be more comfortable with some aspects of place-based approaches consistent with their institutional norms of service delivery, such as collaboration between services and locally tailored interventions, than they are with the more challenging and subtle task of supporting community-led social development processes. While I applaud and admire the Norwegian government for its commitments to address SDH and improve health equity, perhaps the distinction between intervention and empowerment might be of some use to understand the persistence of health inequities,^19^ and strengthen efforts to address them at the local scale. Doing so may require municipal governments and service providers to get out of their comfort zone. Governments or other actors seeking to address health inequities at a local or regional scale can learn from the body of literature on community-based social development and empowerment. This literature critically examines the role of municipal governments in this context,^20^ and discusses a range of principles and strategies that can be employed to genuinely empower and engage community members and organisations including: asset-based development, social capital building, critical awareness, resource mobilisation, and participatory community decision making.^12,21,22^

## Ethical issues

Not applicable.

## Competing interests

Author declares that he has no competing interests.

## Author’s contribution

MF is the single author of the paper.
